# Response of culturable bacteria and their resistance to air purification across compartments

**DOI:** 10.3389/fpubh.2026.1945592

**Published:** 2026-09-28

**Authors:** Laura Mondéjar, Sofya Pozdniakova, Arianna Bruguera, Sílvia Borràs, Sara M Soto

**Affiliations:** 1 ISGlobal, Barcelona, Spain; 2 Faculty of Pharmacy and Food Sciences, University of Barcelona (UB), Barcelona, Spain; 3 AIRLAB, ISGlobal, Barcelona, Spain; 4 Faculty of Medicine and Health Sciences, University of Barcelona (UB), Barcelona, Spain; 5 CIBER Enfermedades Infecciosas (CIBERINFEC), Instituto de Salud Carlos III, Madrid, Spain

**Keywords:** air purification, antimicrobial resistance, bioaerosols, built environment, Indoor air quality, culturable bacteria, occupational exposure

## Abstract

**Introduction:**

The increasing threat of antimicrobial resistance (AMR) in environmental settings, particularly in indoor air, requires robust surveillance and mitigation strategies. This study assessed the taxonomic composition and AMR profiles of human-, airborne- and surface-associated bacteria collected from two indoor environments: office spaces in Valencia (Spain) and school classrooms in Dublin (Ireland).

**Methods:**

Sampling was conducted before and after deployment of portable air purification systems. Samples were analyzed using culture-based methods, MALDI-TOF MS identification, antibiotic susceptibility testing, and PCR detection of resistance genes.

**Results:**

A total of 341 bacteria were recovered. In office air, isolates decreased substantially after air purification (from 41 to 5), with post-intervention samples dominated by *Staphylococcus* spp. and no detectable resistance genes. In contrast, human-associated samples showed reduced diversity, but increased relative abundance of *Staphylococcus* spp. (from 15% to 65%), alongside a higher proportion of non-susceptible isolates (from 20 to 31). In the school environment, airborne isolates and resistance prevalence decreased following intervention (from 102 to 47 and 43% to 21%, respectively), while surface-associated communities showed smaller reductions in total isolates (from 53 to 43), but a notable decline in resistant phenotypes (from 22 to 9), accompanied by shifts in community composition.

**Discussion:**

Overall, air purification was associated with a strong reduction in airborne bacterial recovery and AMR burden, while surface and human-associated microbiota remained more stable. These findings highlight the compartment-specific effects of air purification and support the need for integrated indoor air quality management strategies combining air-targeted interventions with broader environmental control measures to limit AMR dissemination in built environments.

## 1 Introduction

In high-income countries, individuals spend up to 90% of their time indoors making built environments the primary setting for human microbial exposure (1). These enclosed environments harbor diverse microbial communities shaped by outdoor air, human activity, building design, and environmental conditions (2).

Outdoor air is a major contributor to indoor microbial assemblages. Bioaerosols, including bacteria, can efficiently penetrate indoors through ventilation, significantly influencing indoor communities (3). In well-ventilated spaces, indoor microbiomes can resemble that outdoors, although human-associated taxa often accumulate in higher abundance (4).

Human occupancy is a primary driver of indoor airborne microbiota, as individuals continuously release microbial clouds originating from the skin, oral cavity, and respiratory tract, rapidly shaping indoor environments (5). Everyday activities also resuspend settled particles, redistributing microorganisms and increasing airborne concentrations (6). Compared with outdoor air, indoor aerosols are therefore more strongly influenced by biological material shed by occupants, including skin cells, saliva, fecal particles, and respiratory droplets (7). Inhalation remains the principal exposure route, potentially influencing respiratory health and contributing to exposure to resistant microorganisms (8).

Indoor air quality (IAQ) is not only determined by microbial dynamics, but also directly impacts human health. Indoor air pollution is a major global health concern, contributing substantially to morbidity and mortality worldwide (9). Preventive measures such as portable air purifiers have been shown to reduce airborne particulate matter and may contribute to lowering exposure to respiratory pathogens (10). However, excessive disinfection may paradoxically select for resistant organisms (11).

Airborne pathogens, including *Mycobacterium tuberculosis*, *Staphylococcus aureus*, *Haemophilus influenzae*, and fungi such as *Aspergillus fumigatus*, have been repeatedly detected in healthcare settings, alongside viral agents such as influenza, MERS-CoV, SARS-CoV, and SARS-CoV-2 (12). High-throughput DNA sequencing studies confirm that indoor environments are consistently enriched in human-associated bacterial taxa, especially skin-related genera such as *Corynebacterium*, *Streptococcus*, *Staphylococcus*, and *Propionibacterium* (13).

Beyond exposure levels, a critical concern is the circulation of antimicrobial resistance (AMR) in indoor environments. Anthropogenic activities have increased the release of antibiotics, antibiotic-resistant bacteria (ARB), and antibiotic resistance genes (ARGs), elevating environmental levels far above natural baselines (14). The airborne suspension of ARB and ARGs facilitates long-distance transport, crossing environmental boundaries and increasing exposure risks for humans and animals (7, 15, 16).

Indoor environments have also been shown to harbor diverse ARGs, including *tet*, *sul1*, *bla*_TEM_, and *erm* genes, sometimes at levels comparable to or exceeding that outdoors. Such genes have been detected in schools, universities, markets, and even air conditioning systems (17). These findings underscore the role of indoor air as a potential reservoir and dissemination route for AMR.

The objectives of this study were to: (1) characterise the taxonomic composition of culturable airborne bacteria in two distinct indoor environments: office spaces in Valencia (Spain) and school classrooms in Dublin (Ireland), complemented by human samples from the Valencia offices staff and surface samples from the Dublin classrooms; (2) assess the presence and distribution of ARGs among the culturable bacteria recovered from these environments; and (3) evaluate the effectiveness of portable air purifiers as a mitigation strategy by comparing samples collected before and after air purifier deployment to assess changes in microbial composition, resistance phenotypes and ARGs carriage. Overall, this approach aimed to determine whether air purification can serve as an effective preventive measure for improving IAQ and limiting the dissemination of antimicrobial-resistant microorganisms in built environments.

## 2 Materials and methods

### 2.1 Sampling sites

Samples were collected in Dublin (Ireland) and Valencia (Spain) as part of the Horizon Europe project “Digital Twins Enabled Indoor Air Quality Management for Healthy Living” (TwinAIR; GA no. 101057779). The sampling campaign comprised two phases: pre-intervention (November–December 2023) and post-intervention (December 2024–January 2025), the latter conducted following the deployment of air purifiers. In Dublin, sampling was performed at Harold’s Cross Educate Together National School across three indoor environments (Eagle Class, Buzzard Class, and Staff Room). In Valencia, samples were collected from two office spaces at ETRA I + D (Office 1 and 2). The characteristics of the sampled rooms and intervention deployment are summarized in Supplementary Table 1, including room area, maximum occupancy, and number of air purifiers installed. Sampling timelines and intervention deployment were defined by project logistics and site availability.

### 2.2 Human and surface sampling

Human and surface samples were collected using moistened sterile cotton swabs. All participants provided informed consent, and the study protocol “TwinHEALTH” was approved by the Research Ethics Committee of the Hospital del Mar Research Institute (Barcelona, Spain, Reference 2023/11008/I).

In Valencia, a total of 46 human samples were collected from office staff (Supplementary Table 2). During the pre-intervention phase, 12 participants provided nasal and hand swabs (*n* = 24), while 11 participants contributed samples post-intervention (*n* = 22, 1 participant was unavailable).

In Dublin, surface samples were collected from high-contact areas, including classrooms, bathrooms, the kitchen, and common spaces. A total of 48 samples were obtained (pre-intervention: *n* = 18; post-intervention: *n* = 30) (Supplementary Table 2).

### 2.3 Air sampling

Airborne particulate matter with an aerodynamic diameter of less than 10 μm (PM_10_) was collected on sterile micro-quartz fiber filters using two high-volume portable air samplers (Sibata HV-500R, Saitama, Japan) equipped with a PM_10_ inlet, operating at a flow rate of 500 L/min. Sampling was conducted in each room over three consecutive days for 4 hours at the end of each day. Windows and doors were kept closed to ensure consistent indoor conditions.

Following collection, each filter was carefully folded enclosing the exposed surface and stored in sterile bags at −20 °C until transported to the laboratory. Once in the laboratory, filters were handled under biosafety conditions to prevent cross-contamination. Each filter was then sectioned into eight equal parts, individually sealed in sterile bags, and stored at −80 °C for subsequent analysis. Field blanks were included to monitor potential contamination and processed identically. Two portions of each filter were used for culture-based analysis. In Dublin, a total of 15 and 11 filters (including blanks) were collected during the pre- and post-intervention phases, respectively, while 8 filters per phase (including blanks) were collected in Valencia (Supplementary Table 2).

### 2.4 Intervention

Portable AIR+ 300 air purifiers (ZONAIR3D S. L., Barcelona, Spain) were deployed in the study rooms (Supplementary Table 1). Devices were activated between the pre- and post-intervention campaigns, and operated continuously throughout the working day during the post-intervention period.

According to the manufacturer’s specifications, the AIR+ 300 is designed for indoor spaces of 35–50 m^2^ and has a clean air delivery rate (CADR) of 332 m^3^/h. The air purifier incorporate three filtration stages: (1) a G4 pre-filter, for the removal of large particles (PM10), (2) an exclusive gas filter GRSystem™ (ZONEAIR3D), designed to capture gaseous pollutants and reduce their concentrations to comfort levels, and (3) an absolute HEPA filter (U15), which incorporates nanofibers with virucidal properties, for removing microparticles (PM 0.1).

### 2.5 Culture and isolation of bacterial colonies

Samples were cultured on Columbia Nalidixic Acid (CNA) agar (Becton Dickinson (BD), Franklin Lakes, NJ, United States), for Gram-positive bacteria and MacConkey agar (BD), for Gram-negative bacteria. A portion of each filter (blank or sample) was placed in direct contact with the agar surface. Because only limited portions of the filter material were available, duplicate or triplicate cultures could not be performed. Plates were incubated at 35 °C and 30 °C, respectively, for 24 h. Distinct colony morphotypes were selected and subcultured on Columbia agar supplemented with 5% sheep blood (BD). In this work, we focused on culturable bacteria because the study aimed to obtain viable bacterial isolates for subsequent characterization of antimicrobial resistance phenotypes and genotypes, including the ARGs. Culture-independent approaches are being investigated to provide a broader characterization of the microbial community and resistome.

### 2.6 Identification of bacterial isolates by MALDI-TOF MS

Pure isolates were identified by direct transfer using matrix-assisted laser desorption ionization-time of flight mass spectrometry (MALDI-TOF MS) (MALDI Biotyper, Bruker Daltonics GmbH, Bremen, Germany). The database for identification was the reference MBT Compass Library DB-7854 (Bruker Daltonics GmbH). For quality control, *Escherichia coli* ATCC 25922 was used as a reference isolate. After identification, all isolates were stored at −80 °C in skim milk.

### 2.7 Antimicrobial susceptibility testing

The antimicrobial susceptibility profiles of the isolates were evaluated using the Kirby–Bauer disk diffusion method on Mueller–Hinton II (MH) agar plates (BD) or the broth microdilution method, following the guidelines of the European Committee on Antimicrobial Susceptibility Testing (EUCAST) and classified as resistant (R), intermediate (I), or susceptible (S) (18). According to the species, the antibiotics tested were: amikacin (AN), amoxicillin/clavulanic acid (AMC), ampicillin (AMP), aztreonam (ATM), cefepime (FEP), cefoxitin (FOX), cefotaxime (CTX), ceftazidime (CAZ), chloramphenicol (CHL), ciprofloxacin (CIP), clindamycin (CC), erythromycin (E), gentamicin (GM), imipenem (IMI), meropenem (MEM), piperacillin–tazobactam (TZP), rifampicin (RIF), tetracycline (TET), trimethoprim/sulfamethoxazole (SXT). The broth microdilution method, used for selected genera such as *Micrococcus*, *Bacillus*, and *Microbacterium*, was performed using bacterial inocula diluted in cation-adjusted MH broth (BD) and exposed to serial two-fold dilutions of each antimicrobial agent to determine minimum inhibitory concentrations (MICs).

*Escherichia coli* ATCC 25922, *Pseudomonas aeruginosa* ATCC 27853, and *Staphylococcus aureus* ATCC 29213 were used as control strains. For microorganisms without species-specific breakpoints, the breakpoints established for the closest genus or family were used.

### 2.8 Detection of *β*-lactamases and carbapenemases

The isolates non-susceptible (showing full- or intermediate-resistance) to third-generation cephalosporins (3GC) were tested for the CTX-M group enzymes using the NG-Test^®^ CTX-M Multi (NG-Biotech Laboratories, Guipry- Messac, France). This assay detects enzymes from the five major CTX-M groups but does not differentiate the specific variant present. Carbapenem-resistant isolates were further analyzed with the NG-Test^®^ CARBA 5 (NG-Biotech Laboratories) for the detection of the five main carbapenemase enzymes: KPC, OXA-48-like, VIM, IMP, and NDM.

### 2.9 DNA extraction and profiling of antibiotic resistance genes by PCR

DNA extraction was performed using a boiling method as previously described by Mondéjar et al. (19). Briefly, a colony from an overnight culture of each isolate was suspended in 200 μL of Milli-Q water, boiled for 10 min, and centrifuged at 13,000 rpm for 10 min. The resulting supernatant was used as the DNA template for PCR analysis. PCR was performed to detect ARGs including Extended-Spectrum *β*-Lactamases (ESBL) genes (*bla*_SHV_, *bla*_TEM_, *bla*_PER_, *bla*_VEB_, *bla*_GES,_ *bla*_CTX-M-1_, *bla*_CTX-M-9_), AmpC beta-lactamase genes (*bla*_MOX_, *bla*_CIT_, *bla*_FOX_, *bla*_DHA_, *bla*_EBC_), quinolones (*qnrA*, *qnrS*), aminoglycosides (*aph(6)-Id*, *aadA5*, *aph(3″)-lb*, *aac(6′)-lb-cr*), sulfonamides (*sul1*, *sul2*), chloramphenicol (*catI*, *cmIA*), tetracyclines (*tetA*, *tetB*), erythromycin and clindamycin (*ermA*, *ermB*, *ermC*, *mrsA*), rifampicin (*rpoB*) and methicillin-resistant gene (*mecA*). Primer sequences are provided in Supplementary Table 3.

### 2.10 Statistical analysis

Phenotypic antimicrobial resistance was analysed at the isolate level using isolates with at least one interpretable antimicrobial susceptibility testing (AST) result (S, I, or R). The primary endpoint was resistance (R) to at least one tested antibiotic; intermediate susceptibility (I) was retained separately and was not classified as resistant in the primary analysis. Resistance prevalence and exact 95% binomial confidence intervals were calculated for each compartment and intervention phase. Pre/post differences in the overall proportion of resistant isolates were evaluated using Fisher’s exact test, with post/pre risk ratios (RR) and 95% confidence intervals reported as effect estimates. When a zero-cell occurred, a Haldane–Anscombe correction (+0.5 to each cell) was applied only for estimation of the RR and its confidence interval, whereas Fisher’s exact test remained uncorrected. Antibiotic-specific pre/post differences were also assessed using Fisher’s exact test among isolates with an interpretable result for the respective antibiotic, and the resulting *p*-values were adjusted for multiple comparisons using the Benjamini–Hochberg procedure. A secondary analysis considering R and I together as non-susceptible was performed as a sensitivity analysis. Targeted ARG results were summarised descriptively because gene testing was performed on a selected and uneven subset of isolates, precluding formal comparison of ARG prevalence between intervention phases.

## 3 Results

### 3.1 Overview of sampling and isolate recovery

Across both case studies, a total of 136 samples were collected across the two pilot sites, including 42 air filters, and 48 surface and 46 human swabs (Figure 1A). In the office setting, isolates were recovered from human swabs and air samples, whereas in the school setting, air and surface samples were analysed to characterise culturable bacterial communities across different indoor compartments. Culture based analysis yielded a total of 341 culturable bacteria and 3 fungal isolates (recovered incidentally and not included in further analysis) (Supplementary Figure 1A). Bacterial isolates were recovered from human (*n* = 51), air (*n* = 195), and surface (*n* = 96) samples collected before and after the deployment of portable air purifiers. An overview of the sampling effort and isolates recovery from the different sample types is provided in Supplementary Table 2.

**Figure 1:**
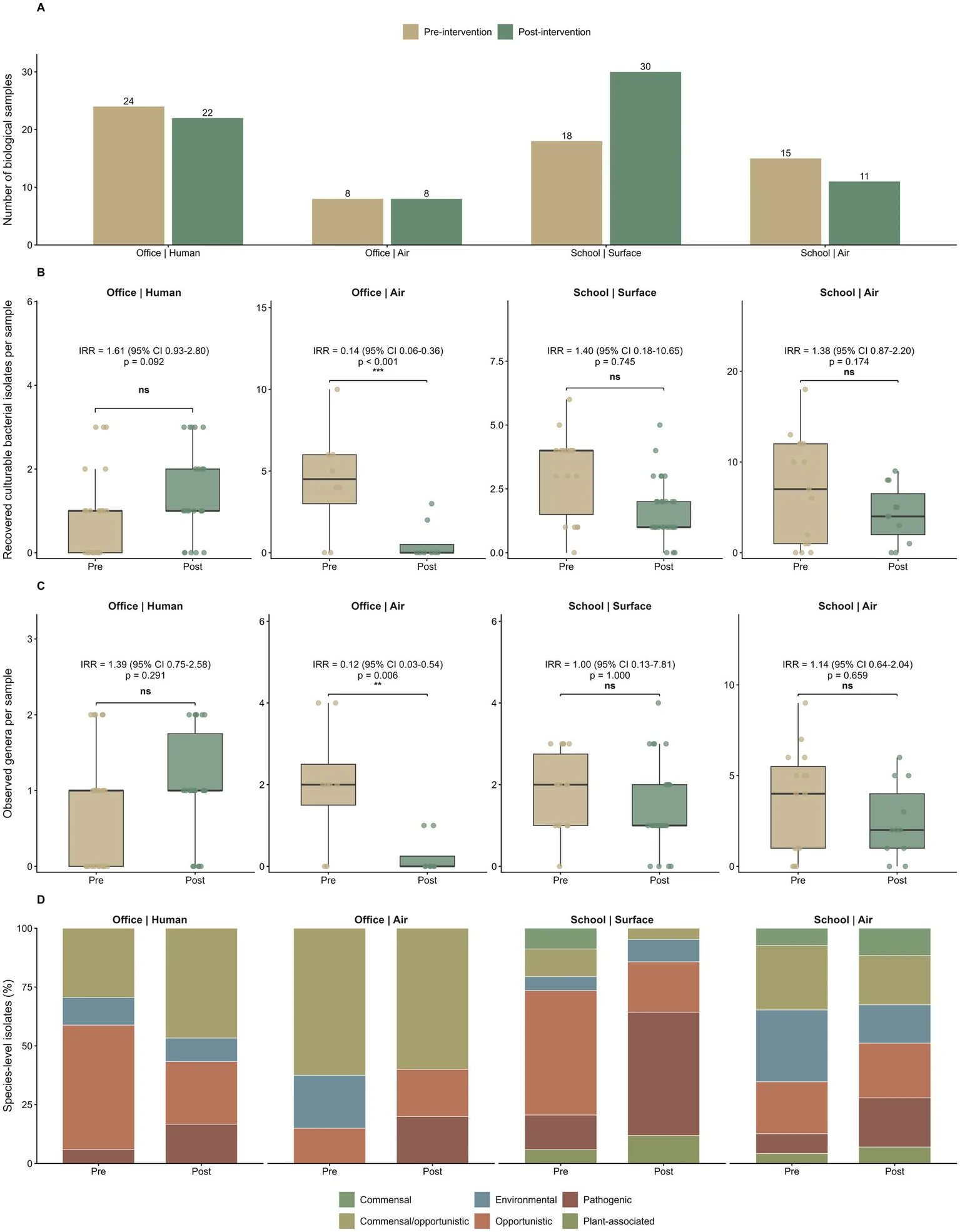
Sampling effort, culturable bacterial recovery, genus richness, and clinical/ecological relevance across intervention phases in the Valencia office and Dublin school. **(A)** Number of biological samples collected during the pre- and post-intervention phases. **(B)** Number of culturable bacterial isolates recovered per sample. **(C)** Observed genus richness per sample; samples without bacterial recovery were assigned a richness of zero. For (B -C), boxes represent the median and interquartile range, with whiskers extending to 1.5 × IQR; pre/post differences were assessed using compartment-specific count regression models, with effect estimates reported as post/pre incidence rate ratios (IRR), 95% confidence intervals (CI), and p-values. **(D)** Relative distribution of species-level bacterial isolates according to curated clinical/ecological relevance categories. Categories represent broad organism associations and do not indicate infection risk. Significance: *** *p* < 0.001, ** *p* < 0.01, * *p* < 0.05, ns, not significant.

Overall, Gram-positive bacteria were slightly more frequently recovered than Gram-negative bacteria, although their relative distribution differed between sample types and intervention phases. Non-spore-forming bacteria predominated across all sample types, whereas spore-forming isolates represented a comparatively small fraction of the cultured bacterial community (Supplementary Figures 1B,C). These broad phenotypic profiles remained generally similar across intervention phases, with no marked restructuring of either Gram classification or spore-forming capacity.

To account for differences in sampling effort between sample types and intervention phases, culturable bacterial recovery and genus richness were additionally analysed at the level of individual biological samples. A significant post-intervention reduction was observed in the Valencia office air samples for both culturable bacterial recovery (IRR = 0.14, 95% CI 0.06–0.36, *p* < 0.001), and observed genus richness (IRR = 0.12, 95% CI 0.03–0.54, *p* = 0.006), while no statistically significant pre/post differences were detected for the other samples (Figure 1B,C; Supplementary Table 4). In particular, genus richness in Valencia human swabs remained broadly comparable between intervention phases, despite a modest increase in isolate recovery after intervention that did not reach statistical significance.

The clinical and ecological relevance profiles of the cultured bacterial isolates also differed between sample types and settings (Figure 1D). In the office samples, isolates were predominantly distributed among commensal/opportunistic, opportunistic, and environmental categories, whereas the pathogenic category represented a comparatively smaller proportion. In contrast, the school samples showed a broader representation of clinical and ecological categories, including commensal, commensal/opportunistic, environmental, opportunistic, pathogenic, and plant-associated organisms, indicating a more heterogeneous clinical/ecological profile among the organisms recovered from the school environment. The clinical/ecological profiles also varied across intervention phases (Figure 1D). In human swabs from the offices, the profile remained broadly similar after the intervention, although the proportion of opportunistic isolates decreased. In office air samples, environmental isolates were no longer detected post-intervention. In school surface samples, the proportions of pathogenic and plant-associated isolates increased, whereas opportunistic isolates decreased after the intervention. In contrast, the clinical/ecological profile of airborne bacterial isolates recovered from the school remained broadly similar between the two phases.

Overall, the cultured bacterial communities comprised both human-associated and environmental taxa, with the strongest intervention-related changes observed in bacterial recovery and genus richness in the office air, and in the relative clinical/ecological composition of surface isolates in the school.

### 3.2 Taxonomic composition and community structure across case studies

In the pre-intervention sampling campaign, the culturable bacterial community recovered from human swabs in the Valencia office exhibited a heterogeneous taxonomic profile. *Pseudomonas* spp. was the most frequently recovered genus followed by *Staphylococcus* spp. with *Acinetobacter* spp., *Moraxella* spp., *Serratia* spp. and other genera represented at lower frequencies (Figures 2A,C). Following air purifier deployment, *Staphylococcus* spp. Became the dominant genus increasing up to 64.5%, while non-staphylococcal genera represented a smaller proportion of the cultured community and the genus distribution became less even (Figures 2A,C). At species level, this shift was characterised particularly by increased representation of *S. epidermidis* (Figures 2B,D).

**Figure 2:**
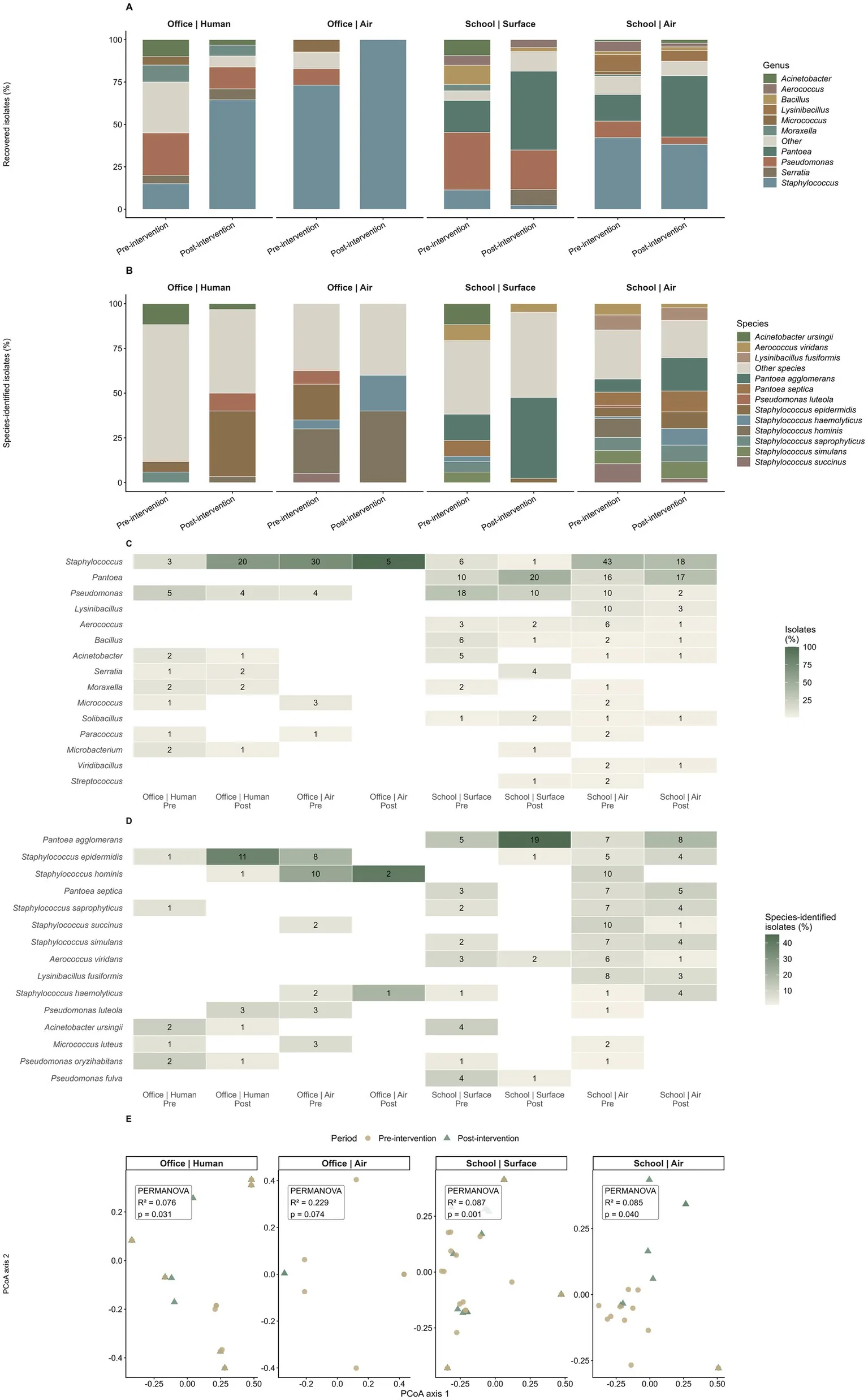
Taxonomic composition and antimicrobial resistance profiles of culturable bacterial communities recovered from the Valencia office and Dublin school before and after portable air purifier deployment. **(A)** Genus-level composition of cultured bacterial isolates from human swabs and air samples in the office, and surface and air samples in the school, with the most frequently recovered genera shown individually and remaining genera grouped as “Other.” **(B)** Species-level composition of isolates identified to species level, showing the most frequently recovered species and remaining species as “Other species.” (C -D) Distribution of dominant bacterial genera **(C)** and species **(D)** across sample types and intervention phases. Cell shading represents the relative proportion of recovered isolates, and numbers indicate isolate counts. **(E)** PCoA of genus-level community composition based on presence/absence Jaccard dissimilarity. Pre/post differences were assessed separately for each sampling compartment using PERMANOVA, with *R*² and *p*-values shown in each panel. Samples without bacterial recovery were excluded from the beta-diversity analysis.

A similar increase in taxonomic dominance was observed in office air samples. Before intervention, *Staphylococcus* spp. already represented the predominant genus (73.2%, *n* = 30), but *Pseudomonas* spp. (9.8%, *n* = 4), *Micrococcus* spp. (7.3%, *n* = 3), and several additional genera were also recovered. Following intervention, all five identified airborne bacterial isolates belonged to *Staphylococcus* spp. (100%), resulting in a markedly more restricted genus-level profile (Figures 2A,C). At species level, the small post-intervention airborne assemblage comprised several *Staphylococcus* spp. (Figures 2B,D).

In the Dublin school, before intervention, *Pseudomonas* spp. was the most frequently recovered genus (34.0%, *n* = 18) from the surface samples, followed by *Pantoea* spp. (18.9%, *n* = 10), *Staphylococcus* spp. and *Bacillus* spp. (both 11.3%, *n* = 6) (Figures 2A,C). The remaining isolates were distributed across several lower-frequency genera, resulting in a comparatively broad genus-level profile. Following intervention, *Pantoea* spp. increased to 46.5% (*n* = 20), producing a less even genus distribution dominated by *Pantoea*. *Acinetobacter* and *Moraxella*, recovered before intervention, were no longer detected, whereas genera including *Serratia, Microbacterium* and *Enterobacter* were represented in the post-intervention samples (Figures 2A,C). At species level, *Pantoea agglomerans* became a prominent component of the post-intervention surface community (19 isolates compared with 5 before intervention), whereas *P. septica* and several other species showed phase-specific distributions, consistent with substantial compositional turnover between sampling periods (Figures 2B,D).

Airborne isolates from the Dublin school also showed a heterogeneous taxonomic composition. Before intervention, *Staphylococcus* spp. represented 42.2% of the cultured isolates (*n* = 43), followed by *Pantoea* spp. (15.7%, *n* = 16), *Pseudomonas* spp. and *Lysinibacillus* spp. (both 9.8%, *n* = 10), with *Aerococcus*, *Bacillus* and several additional environmental genera detected at lower frequencies (Figures 2A,C). Post-intervention air remained dominated by *Staphylococcus* spp. (38.3%, *n* = 18), while the relative representation of *Pantoea* spp. increased to 36.2% (*n* = 17) and *Pseudomonas* spp. decreased to 4.3% (*n* = 2). Thus, the post-intervention airborne profile became concentrated primarily around *Staphylococcus* and *Pantoea*, while several genera that contributed to the broader pre-intervention profile were recovered less frequently or were no longer represented. Multiple staphylococcal and environmental species remained represented across both sampling phases, although their relative distributions changed between periods (Figures 2B,D). At species level, *P. agglomerans* increased from 7 pre-intervention isolates to 8 post-intervention isolates, while the increased genus-level contribution of *Pantoea* also reflected representation of additional *Pantoea* spp. (Figure 2D).

Genus-level community structure was further assessed using presence/absence-based Jaccard dissimilarity (Figure 2E). PERMANOVA provided the strongest evidence of a pre/post difference in school surface communities (*R*^2^ = 0.087, *p* = 0.001). Nominal pre/post differences were also observed for office human samples (*R*^2^ = 0.076, *p* = 0.031) and school air samples (*R*^2^ = 0.085, *p* = 0.040), whereas the office airborne community did not reach statistical significance despite a larger estimated effect size (*R*^2^ = 0.229, *p* = 0.074) (Supplementary Table 5). After Benjamini-Hochberg correction across the four compartment-specific comparisons, only the school surface comparison remained statistically significant (adjusted *p* = 0.004); the adjusted *p* values for office human and school air were both approximately 0.053, and that for office air was 0.074. Accordingly, although the ordinations show visual pre/post shifts in several compartments, only the school surface community provided statistically supported evidence of a compositional difference after multiple-testing correction.

### 3.3 Antimicrobial resistance profiles across case studies

Phenotypic antimicrobial resistance was evaluated at the isolate level among isolates with interpretable antimicrobial susceptibility testing (AST) results, using resistance (R) to at least one tested antibiotic as the primary endpoint. Intermediate susceptibility (I) was retained separately and was not classified as resistance in the primary analysis. In human samples from the offices, the proportion of resistant isolates increased from 2/17 (11.8%) before intervention to 6/31 (19.4%) after intervention. However, this difference was not statistically significant (RR = 1.65, 95% CI 0.37–7.28, *p* = 0.694). In office air, resistant isolates decreased from 9/37 (24.3%) before intervention to 0/5 after intervention, although this difference was also not statistically significant (RR = 0.33, 95% CI 0.02–5.01, *p* = 0.567). The highest pre-intervention prevalence was observed in school surface isolates, where resistance decreased from 22/51 (43.1%) to 9/43 (20.9%) following intervention (RR = 0.49, 95% CI 0.25–0.94, *p* = 0.028). In school air, resistance was detected in 7/29 isolates (24.1%) before and 8/45 isolates (17.8%) after intervention, with no significant difference between phases (RR = 0.74, 95% CI 0.30–1.81, *p* = 0.562) (Figure 3A; Supplementary Table 6). Thus, among the four compartment-level comparisons, only the reduction observed in school surface isolates reached statistical significance (unadjusted *p* < 0.05).

**Figure 3:**
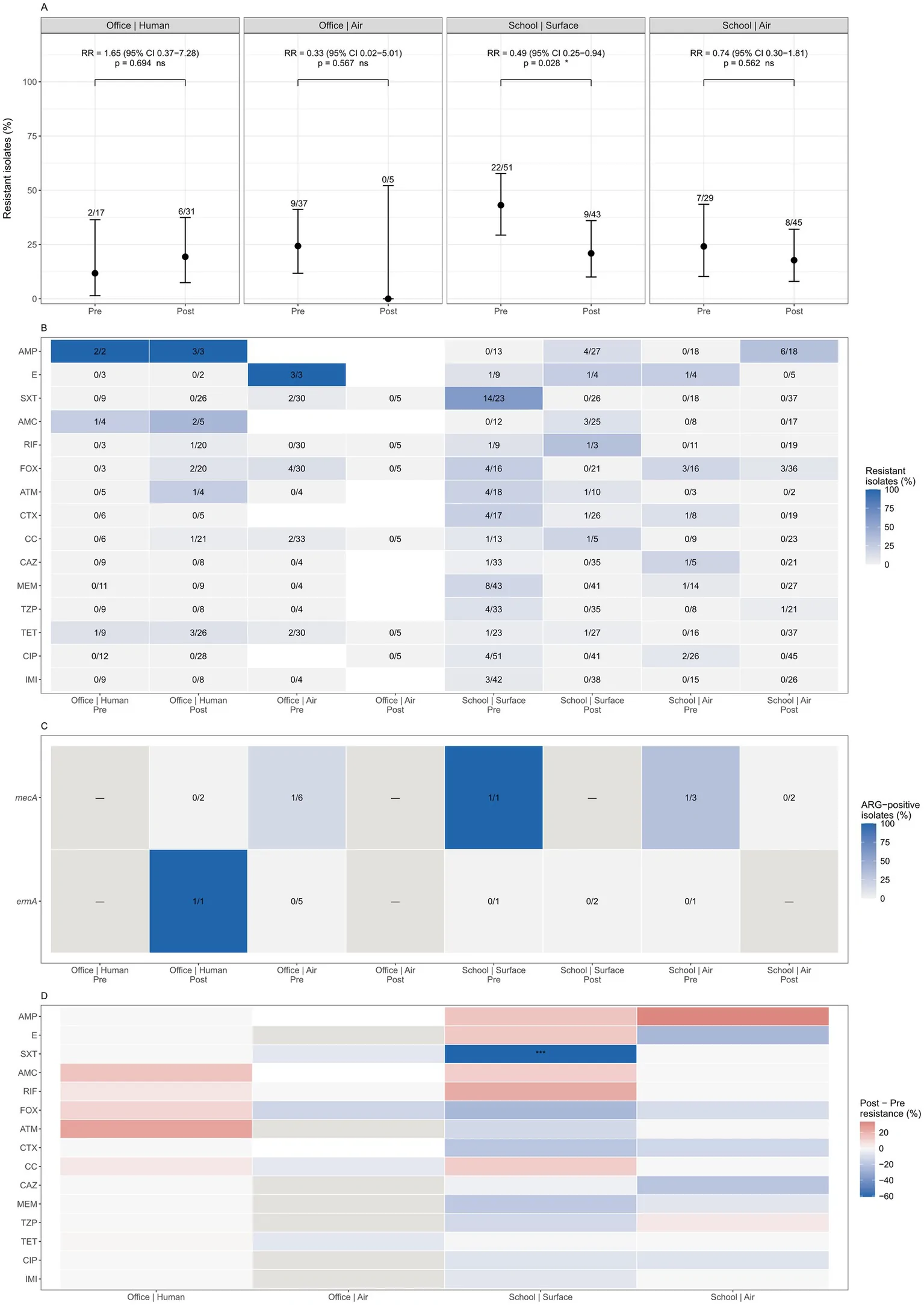
Phenotypic antimicrobial resistance and targeted antimicrobial resistance gene detection among culturable bacterial isolates across intervention phases. **(A)** Prevalence of phenotypic resistance among isolates with interpretable antimicrobial susceptibility testing results. Resistance was defined as resistance **(R)** to at least one tested antibiotic; intermediate **(I)** isolates were not considered resistant. Points represent the proportion of resistant isolates, with exact 95% binomial confidence intervals (CI); values indicate resistant/tested isolates. Pre/post differences within each compartment were assessed using Fisher’s exact test, with post/pre risk ratios (RR) and 95% CI as effect estimates. **(B)** Antibiotic-specific phenotypic resistance. Cell shading represents the percentage of resistant isolates, with resistant/tested counts shown within cells; only antibiotics with at least one resistant isolate are shown. **(C)** ARG detection among tested isolates. Cell shading represents the percentage of ARG-positive isolates, with positive/tested counts shown within cells; grey cells indicate genes that were not tested. Only detected genes are shown. **(D)** Changes in antibiotic-specific resistance prevalence between post- and pre-intervention phases. Cell shading represents the post-minus-pre difference in the percentage of resistant isolates. Comparisons were performed using Fisher’s exact test for each compartment -antibiotic combination, with Benjamini -Hochberg correction for multiple testing. Significance is indicated as *** adjusted *p* < 0.001, ** adjusted *p* < 0.01, and * adjusted *p* < 0.05.

Antibiotic-specific resistance profiles differed markedly between sample types and study sites (Figure 3B; Supplementary Table 7–10). In office human isolates, resistance was detected for a limited number of antibiotics. Ampicillin resistance was observed in all isolates tested for this antibiotic, although the number of isolates tested was small (2/2 pre-intervention and 3/3 post-intervention). Resistance to amoxicillin-clavulanate was detected in 1/4 and 2/5 tested isolates before and after intervention, respectively, while resistance to tetracycline remained infrequent (1/9 pre and 3/26 post). Additional post-intervention resistance events included cefoxitin, aztreonam, rifampicin, and clindamycin. At isolate level, resistant phenotypes were observed among both Gram-negative bacteria and *Staphylococcus epidermidis*, including individual *S. epidermidis* isolates resistant to multiple tested agents (Supplementary Table 7).

In office air, resistance before intervention was detected to erythromycin (3/3 tested isolates), cefoxitin (4/30), trimethoprim/sulfamethoxazole (2/30), tetracycline (2/30), and clindamycin (2/33), whereas no resistant phenotype was detected among the five post-intervention airborne isolates for any antibiotic with interpretable testing results (Figure 3B). The pre-intervention resistance events were largely associated with *Micrococcus luteus* and coagulase-negative *Staphylococcus* spp., while the tested *Pseudomonas* isolates showed no resistant phenotype under the R-only definition (Supplementary Table 8). Because only five isolates were recovered post-intervention, the absence of detected resistance in this group should be interpreted together with the small number of isolates tested and the non-significant compartment-level comparison.

The broadest phenotypic resistance profile was observed among school surface isolates before intervention (Figure 3B; Supplementary Table 9). Resistance to trimethoprim/sulfamethoxazole was particularly frequent (14/23; 60.9%), accompanied by resistance to meropenem (8/43), cefoxitin (4/16), aztreonam (4/18), cefotaxime (4/17), piperacillin–tazobactam (4/33), ciprofloxacin (4/51), and imipenem (3/42). These phenotypes were distributed across several taxa, including *Aerococcus viridans*, *Bacillus* spp., *Pantoea* spp., *Pseudomonas* spp., and staphylococci. Following intervention, resistance to several of these antibiotics was reduced or no longer detected, including trimethoprim/sulfamethoxazole (0/26), cefoxitin (0/21), meropenem (0/41), piperacillin–tazobactam (0/35), ciprofloxacin (0/41), and imipenem (0/38), whereas low-frequency resistance remained detectable for several other agents. After Benjamini–Hochberg correction of the antibiotic-specific comparisons, only the reduction in trimethoprim/sulfamethoxazole resistance remained statistically significant, corresponding to a decrease of approximately 61% (BH-adjusted *p* < 0.001; Figure 3D).

Resistance among school airborne isolates was less concentrated around a single antibiotic and was distributed across several taxa and antimicrobial classes (Figure 3B; Supplementary Table 10). Before intervention, resistant isolates were detected for erythromycin (1/4), cefoxitin (3/16), cefotaxime (1/8), ceftazidime (1/5), meropenem (1/14), and ciprofloxacin (2/26). Post-intervention resistance was mainly observed for ampicillin (6/18), cefoxitin (3/36), and piperacillin–tazobactam (1/21). The isolate-level profiles indicate contributions from both Gram-positive and Gram-negative taxa, including staphylococci, *Aerococcus*, *Pantoea*, and related environmental organisms (Supplementary Table 10). However, none of the antibiotic-specific pre/post differences in school air remained statistically significant after correction for multiple comparisons (Figure 3D).

Targeted ARG screening identified two resistance genes with positive results in the revised dataset, *mecA* and *ermA* (Figure 3C). *mecA* was detected among tested pre-intervention isolates from office air (1/6), school surfaces (1/1), and school air (1/3), whereas it was not detected among tested post-intervention office human (0/2) or school air isolates (0/2). *ermA* was only detected in one post-intervention office human isolate (1/1 tested) and was not detected in the other tested compartment–period samples (Figure 3C). Because ARG screening was targeted and the numbers tested for individual genes were small and uneven across sample types, these results were interpreted descriptively rather than subjected to formal pre/post inference.

Overall, phenotypic resistance varied substantially between sample types and sites. The clearest reduction was observed in school surface isolates, both in the overall prevalence of resistant isolates and specifically for trimethoprim/sulfamethoxazole resistance. In office air, no phenotypically resistant isolate was recovered after intervention, but the small number of post-intervention isolates and the non-significant overall comparison preclude a strong intervention-effect conclusion. Changes in office human and school air isolates were likewise not statistically supported. Targeted ARG screening revealed a limited number of detected resistance determinants among the isolates tested.

### 3.4 Cross-compartment and cross-setting comparison of indoor bacterial communities

#### 3.4.1 Human and air bacterial communities across intervention phases

Across both sampling phases, several bacterial genera were detected in both human and airborne samples, indicating partial overlap between human-associated and airborne culturable bacterial communities (Figure 4A). During the pre-intervention phase, four species were shared between human swabs and air: *Staphylococcus epidermidis*, *S. warneri*, *Micrococcus luteus*, and *Paracoccus yeei*. Following air purifier deployment, the composition of the shared fraction changed, with *S. hominis* and *S. aureus* recovered from both compartments, while the species shared before intervention were no longer jointly detected (Figure 4A).

**Figure 4:**
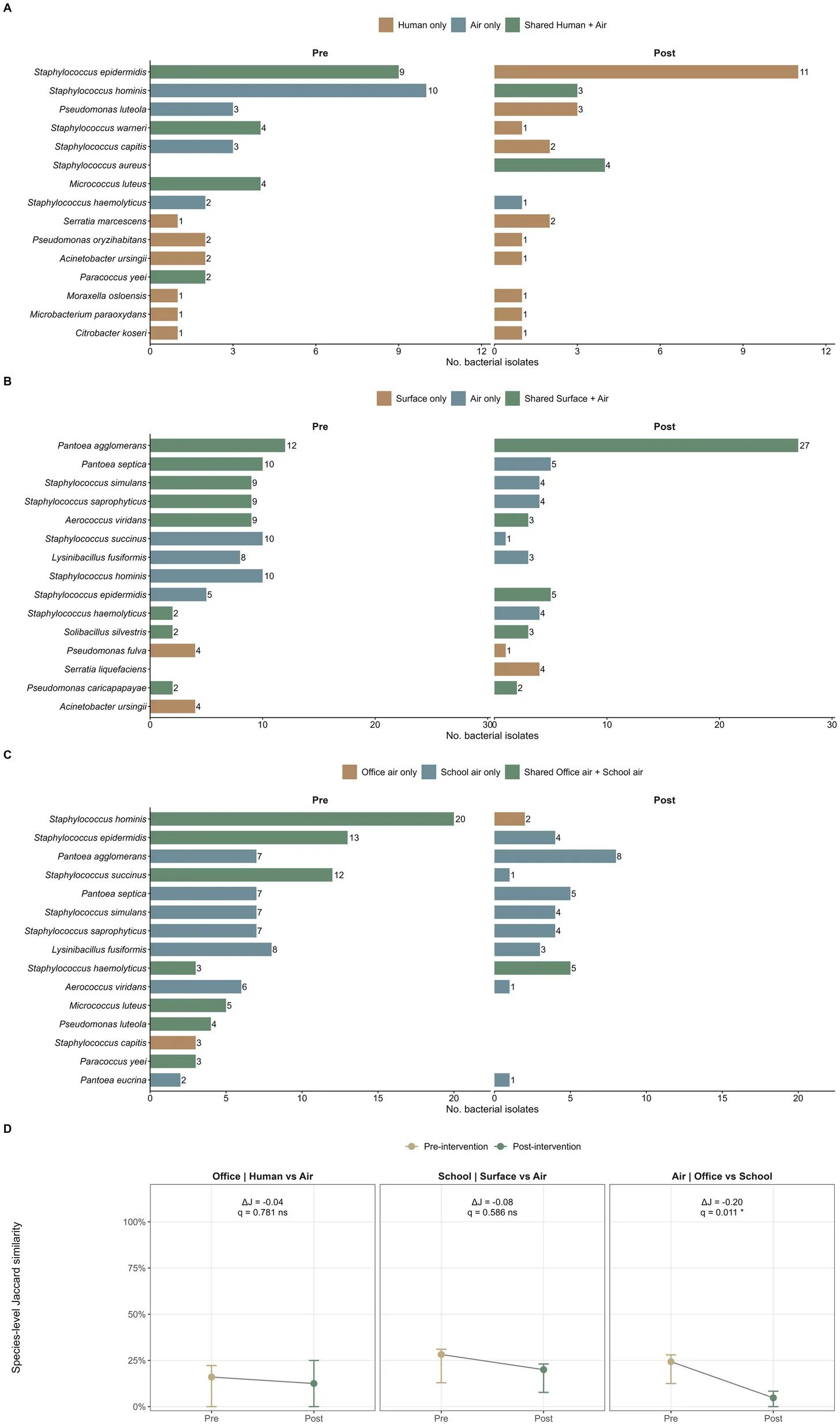
Cross-compartment and cross-setting overlap of culturable bacterial communities across intervention phases. **(A)** Species-level overlap between human swabs and air samples from the office before and after intervention. **(B)** Species-level overlap between surface swabs and air samples from the school. **(C)** Cross-setting comparison of airborne species recovered from the office and school. In panels A -C, dominant cultured species are classified as shared between paired compartments/settings or specific to either group; bar lengths indicate the total number of bacterial isolates corresponding to each species. **(D)** Species-level Jaccard similarity based on the full species pool for each comparison. Points represent observed Jaccard similarity, with 95% bootstrap confidence intervals based on resampling biological samples within each compartment and intervention phase. Pre/post changes were assessed using sample-level permutation tests, with Benjamini -Hochberg correction across the three comparisons; ΔJ represents the post-intervention minus pre-intervention change in Jaccard similarity. Species-overlap analyses describe compositional similarity and should not be interpreted as evidence of microbial transmission or source directionality. *q* < 0.05; ns, not significant.

At the community level, species-level Jaccard similarity between human and airborne communities decreased slightly from 0.160 before intervention to 0.125 after intervention (ΔJ = −0.04). However, this change was not statistically significant after correction for multiple comparisons (BH-adjusted *q* = 0.781; Figure 4D). Thus, human–air compositional overlap remained limited across both sampling phases, with turnover in the identity of the species shared between compartments rather than a statistically supported reduction in overall overlap.

#### 3.4.2 Surface and air bacterial communities across intervention phases

In the Dublin school, species-level overlap was observed between surface and airborne communities before intervention (Figure 4B). Shared species included *Pantoea agglomerans*, *P. septica*, *Staphylococcus simulans, S. saprophyticus*, *Aerococcus viridans*, *S. haemolyticus*, *Solibacillus silvestris*, and *Pseudomonas caricapapayae*. Following intervention, several species remained represented in both sample types, including *P. agglomerans, A. viridans, S. epidermidis, S. silvestris,* and *P. caricapapayae*, while other species were detected exclusively in either surface or air samples (Figure 4B).

Species-level Jaccard similarity between surface and airborne communities decreased from 0.282 before intervention to 0.200 after intervention (ΔJ = −0.08), although this difference was not statistically significant after Benjamini–Hochberg correction (*q* = 0.586; Figure 4D). These findings therefore indicate persistent, but dynamic compositional overlap between surface-associated and airborne culturable bacterial communities rather than a statistically supported intervention-related reduction in overlap.

#### 3.4.3 Cross-setting comparison of airborne bacterial communities

Airborne communities from the Valencia office and Dublin school also showed partial species-level overlap before intervention (Figure 4C). Shared species included *S. hominis*, *S. epidermidis*, *S. succinus*, *S. haemolyticus*, *M. luteus*, and *Pseudomonas luteola*, whereas the Dublin school contained a larger set of setting-specific airborne species, including *P. agglomerans*, *P. septica*, *S. simulans*, *S. saprophyticus*, *Lysinibacillus fusiformis*, and *A. viridans* (Figure 4C). Following intervention, clear divergence between environments was observed. Office air contained a restricted species set, whereas school air retained a broader range of setting-specific species, and relatively few species remained shared between the two environments (Figure 4C). This divergence was supported quantitatively by a decrease in species-level Jaccard similarity from 0.243 before intervention to 0.048 after intervention (ΔJ = −0.20), which remained statistically significant after correction for multiple comparisons (BH-adjusted *q* = 0.011; Figure 4D). Thus, unlike the within-setting human–air and surface–air comparisons, the cross-setting airborne comparison showed a statistically supported reduction in compositional similarity following intervention.

## 4 Discussion

The indoor environment represents a dynamic interface where human-associated, airborne, and surface microbiota converge, creating conditions that can support microbial persistence and the presence of AMR (20, 21). Factors such as low humidity, cleaning practices, and continuous microbial shedding from occupants shape these communities and may favor the maintenance and exchange of resistance determinants (22). By comparing two contrasting indoor environments such as offices in Valencia and a school in Dublin, before and after air purifier deployment this study provides insight into how air-targeted interventions influence microbial composition and AMR patterns across compartments.

A consistent pattern across both settings was the compartment-specific response to intervention. The strongest reduction in culturable bacterial recovery and genus richness was observed in office air, whereas no significant changes in these sample-level endpoints were detected in office human swabs or in school surface and air samples. At the community-composition level, however, school surface samples showed a significant pre/post difference after multiple-testing correction. Airborne bacterial communities showed the strongest reduction in both diversity and AMR burden, whereas surface-associated communities were comparatively more stable. This supports the interpretation that air purification primarily affects the airborne fraction of the indoor microbiome, while surfaces may represent more persistent microbial reservoirs (23). However, the present data do not directly demonstrate whether these surfaces function as stable reservoirs. The differing responses between the office and school environments further suggest that occupancy, activity, and environmental complexity modulate intervention outcomes (4, 6).

In the office setting, air purification was associated with a marked simplification of the airborne microbiota, with *Staphylococcus* spp. remaining as the dominant and ultimately exclusive detectable genus. This pattern is consistent with previous studies showing that human occupancy is a major contributor to indoor airborne bacteria, particularly skin-associated taxa (6). Following intervention, the reduction of non-human-associated genera in air coincided with a relative increase in human-associated taxa in the airborne compartment, suggesting a shift toward microbiota more directly linked to occupants (4, 5). Given that only five airborne bacterial isolates were recovered post-intervention, this pattern should be interpreted as a restricted taxonomic profile rather than selective removal of specific microbial sources.

In parallel, human-associated samples showed a relative increase in *Staphylococcus* spp. and no significant change in genus richness. While this pattern may reflect decreased contribution from environmental taxa, it should be interpreted cautiously given the limited sample size (24, 25). The observed increase in resistant isolates among human-associated bacteria was not statistically significant and therefore should not be interpreted as a direct intervention effect. The resistance profiles observed in *S. epidermidis* are consistent with previous reports describing AMR in coagulase-negative staphylococci (26, 27), including the presence of *ermA*, associated with macrolide–lincosamide resistance (28).

In contrast, the airborne compartment in the office environment showed no phenotypically resistant isolates among the five post-intervention isolates tested. Resistant staphylococci and associated ARGs identified prior to intervention were not detected among the limited post-intervention isolates tested. However, the overall compartment-level comparison was not statistically significant and ARG testing was sparse and uneven (29–31).

The school environment exhibited a more complex and resilient microbial structure. Surface-associated communities remained diverse and dominated by environmentally associated genera, while airborne communities retained moderate diversity without a significant reduction in sample-level isolate recovery or genus richness. The persistence of shared genera across air and surfaces is consistent with well-established resuspension processes linking indoor compartments (32), although the present overlap analysis does not demonstrate source direction or resuspension directly.

Following intervention, shifts in community composition were observed, including an increased relative abundance of environmental taxa such as *Pantoea* spp. and the disappearance of others. *Pantoea* spp. are widely distributed environmental bacteria commonly associated with plant material and indoor surfaces (33–36), and their persistence is consistent with their ecological versatility. These changes suggest that air purification may alter community structure.

HEPA filtration is expected to reduce airborne bacteria primarily through physical particle-capture mechanisms, including interception, inertial impaction, and diffusion. HEPA filters are highly efficient at removing airborne particles, with a nominal efficiency of ≥99.97% for particles of 0.3 μm, and most bacterial cells are within the size range effectively captured by these mechanisms. Consequently, the removal of airborne resistant bacteria is likely to result from their physical retention by the filter, although this mechanism was not directly assessed in the present study. Importantly, surface-associated microbiota appeared less responsive to the intervention, reinforcing their role as stable reservoirs within the indoor environment (37).

With respect to AMR, the clearest change was observed in Dublin surface isolates. Overall phenotypic resistance decreased from 43.1 to 20.9%, representing the only compartment-level comparison with an unadjusted *p* < 0.05. At the antibiotic-specific level, only the reduction in trimethoprim/sulfamethoxazole resistance remained statistically significant after Benjamini–Hochberg correction. Several resistant taxa detected prior to intervention were no longer observed, and the number of resistant isolates decreased substantially. Non-susceptibility patterns observed in *Pseudomonas* spp. are consistent with previously reported resistance profiles in environmental and clinical isolates (38, 39). Moreover, the disappearance of methicillin-resistant staphylococci following intervention was notable, as these organisms are recognized opportunistic pathogens with clinically relevant resistance traits (40). In school air, resistant isolates persisted across both phases, and neither the overall resistance prevalence nor antibiotic-specific pre/post differences were statistically significant after correction. However, resistant organisms persisted in both compartments, indicating that air purification alone did not fully eliminate AMR from this indoor environment.

Across both case studies, a key observation was the reduction in overlap between compartments following intervention, particularly between air and other sample types. Prior to intervention, shared taxa and resistance profiles suggested connectivity between compartments. Post-intervention, this overlap decreased, but the reductions in office human–air and school surface–air Jaccard similarity were not statistically significant. In contrast, the airborne communities from offices and the school became significantly less similar after intervention. However, as direct transmission was not measured, these findings should be interpreted as patterns of association rather than evidence of causal pathways. Moreover, the findings are based on one office building and one school and may therefore not be representative of other building types, climates, occupancy patterns, or ventilation systems. Further studies across a broader range of indoor environments and geographical settings are needed to assess the generalizability of these findings.

Bioaerosol dynamics in indoor environments are influenced not only by direct airborne emissions, but also by deposition and subsequent resuspension from indoor surfaces. Human activity, movement, cleaning and ventilation can promote the transfer of previously deposited particles back into the air, contributing to temporal variability in airborne microbial communities. The species overlap observed between air and surface samples in the Dublin school is compatible with such dynamic exchange between compartments. However, the present study did not directly measure deposition or resuspension rates, and therefore these processes cannot be inferred mechanistically from the observed taxonomic overlap alone.

From an applied perspective, these results highlight that air purification can effectively reduce airborne culturable isolate recovery and AMR burden, under some indoor conditions, as observed in the Valencia office, but the effect was not consistent across all settings or compartments. Thus, air purification should be considered a complementary IAQ control measure, rather than a standalone intervention. In hospitals, air purification could be used alongside established infection-prevention measures, including adequate ventilation, environmental cleaning, hand hygiene, and appropriate isolation practices, rather than as a replacement for them. In offices and schools, air purification may similarly represent one component of a broader strategy for managing IAQ and microbial exposure. Its potential value may be greatest when integrated with adequate ventilation, routine cleaning, and appropriate occupancy and maintenance practices. While the airborne fraction is highly responsive in the Valencia office, surface-associated communities remain comparatively resilient in terms of isolate recovery, although their composition changed significantly in the Dublin school, and may continue to contribute to indoor microbial dynamics. This suggests that effective IAQ management strategies should integrate both air-targeted interventions and surface hygiene measures.

Overall, decisions regarding the deployment of air purifiers should consider the specific characteristics of the setting, including ventilation performance, room occupancy, device capacity, maintenance requirements, and the potential contribution of both airborne and surface-associated microbial reservoirs. Further longitudinal and intervention-based studies are needed to determine whether the observed microbial changes translate into reductions in infection transmission or clinically relevant AMR outcomes.

## 5 Conclusion

Air purification was associated with a substantial reduction in airborne microbial diversity and AMR burden, whereas effects on surface-associated communities were more limited. This differential response across compartments suggests that indoor microbial dynamics may be shaped by both environmental reservoirs and human-associated inputs. These findings are consistent with a model in which air purification may reduce airborne microbial load and AMR exposure, while having more limited effects with microbial persistence on indoor surfaces. Further, integrated strategies addressing both air and surface compartments may be needed to effectively mitigate indoor AMR and microbial exposure.

## 6 Study limitations

This study has several limitations that should be considered when interpreting the findings. First, the analysis relied exclusively on culture-dependent methods and therefore, captured only a fraction of the total microbial community. This approach may bias detection toward fast-growing and stress-tolerant organisms and may underestimate the overall microbial diversity and resistome. Therefore, the observed microbial diversity and ARG profiles should be interpreted as representative of the culturable fraction rather than the entire microbial community. Culture-independent approaches, such as metagenomic or 16S rRNA amplicon sequencing, could provide a more comprehensive characterisation of microbial diversity and resistome. Second, the number of isolates varied across compartments and sampling phases, limiting the ability to perform robust quantitative comparisons and increasing sensitivity to compositional effects. Third, the study design does not allow direct assessment of microbial transmission or source attribution and observed overlaps between compartments should not be interpreted as evidence of direct exchange. Fourth, the pre- and post-intervention campaigns were conducted approximately 1 year apart, and ventilation rates, occupancy patterns, and cleaning practices were not systematically quantified, which may have introduced additional variability into the observed differences. Finally, the findings are based on two specific indoor environments and may not be directly generalizable to other building types, climatic conditions, occupancy patterns or ventilation systems.

## Data Availability

The raw data supporting the conclusions of this article will be made available by the authors, without undue reservation.
