# Supplementary material for: Response of culturable bacteria and their resistance to air purification across compartments
Source: Front Public Health. 2026 Sep 28;14:1945592. doi: 10.3389/fpubh.2026.1945592 (PMC13655089; doi:10.3389/fpubh.2026.1945592)
Supplement: Supplementary file 1 [file Data_Sheet_1.docx]

Supplementary Material

# 1 Supplementary Figures and Tables

## 1.1 Supplementary Figures

**Supplementary Figure 1. Culture-based bacterial isolate recovery and broad phenotypic characteristics across study sites and intervention phases.** (A) Total number of cultured bacterial isolates recovered before and after intervention. (B) Relative distribution of Gram-positive, Gram-negative, and isolates with unknown Gram classification among species-level bacterial isolates. (C) Relative distribution of spore-forming, non-spore-forming, and isolates with unknown spore-forming capacity among species-level bacterial isolates.

## 1.2 Supplementary Tables

| Location | Pilot site | Room | Room area (m^2^) | Maximum occupancy (persons) | No. of air purifiers |
| --- | --- | --- | --- | --- | --- |
| Valencia, Spain | Office | Office 1 | 75 | 15 | 2 |
|  |  | Office 2 | 50 | 15 | 2 |
| Dublin, Ireland | School | Eagle Class | 50 | 25 | 1 |
|  |  | Buzzard Class | 50 | 8 | 1 |
|  |  | Staff Room | 40 | 17 | 1 |

**Supplementary Table 1.** Characteristics of study rooms and air purifier deployment.

| Location | Pilot site | Sample type | Sampling phase | No. of samples | No. of positive samples | Sampling method | Sampling volume (m^3^) | Total bacterial isolates | Gram-positive bacteria | Gram-negative bacteria | Spore forming bacteria | Fungi |
| --- | --- | --- | --- | --- | --- | --- | --- | --- | --- | --- | --- | --- |
| Valencia, Spain | Office | Human | Pre | 24 | 13 | Swab | - | 19 | 7 | 12 | 0 | 1 |
|  |  |  | Post | 22 | 17 | Swab | - | 31 | 21 | 10 | 0 | 0 |
|  |  | Air | Pre | 8 | 6 | Filter | 119.9 | 41 | 35 | 6 | 0 | 0 |
|  |  |  | Post | 8 | 2 | Filter | 119.9 | 5 | 5 | 0 | 0 | 0 |
| Dublin, Ireland | School | Surface | Pre | 18 | 17 | Swab | - | 53 | 18 | 35 | 7 | 0 |
|  |  |  | Post | 30 | 27 | Swab | - | 43 | 8 | 35 | 3 | 2 |
|  |  | Air | Pre | 15 | 12 | Filter | 119.9 | 102 | 70 | 32 | 15 | 0 |
|  |  |  | Post | 11 | 9 | Filter | 119.9 | 47 | 25 | 22 | 6 | 0 |
|  |  |  |  | **136** | **103** |  |  | **341** | **189** | **152** | **31** | **3** |

**Supplementary Table 2.** Summary of sampling campaigns, sample characteristics, and microbial isolates recovered from the different types of samples before (pre) and after (post) air purifier deployment.

**Supplementary Table 3.** Primers used for the detection of antibiotic resistance genes by PCR.

|  | Primer name | Sequence (5’->3’) | Annealing temp. (°C) | Product size (bp) | Reference |
| --- | --- | --- | --- | --- | --- |
| Beta-lactams | *bla*_MOX_-F | GCTGCTCAAGGAGCACAGGAT | 64 | 520 | (41) |
|  | *bla*_MOX_-R | CACATTGACATAGGTGTGGTGC |  |  |  |
|  | *bla*_CIT_-F | TGGCCAGAACTGACAGGCAAA | 64 | 462 | (41) |
|  | *bla*_CIT_-R | TTTCTCCTGAACGTGGCTGGC |  |  |  |
|  | *bla*_DHA_-F | AACTTTCACAGGTGTGCTGGGT | 64 | 405 | (41) |
|  | *bla*_DHA_-R | CCGTACGCATACTGGCTTTGC |  |  |  |
|  | *bla*_EBC_-F | TCGGTAAAGCCGATGTTGCGG | 64 | 302 | (41) |
|  | *bla*_EBC_-R | CTTCCACTGCGGCTGCCAGTT |  |  |  |
|  | *bla*_FOX_-F | AACATGGGGTATCAGGGAGATG | 64 | 190 | (41) |
|  | *bla*_FOX_-R | CAAAGCGCGTAACCGGATTGG |  |  |  |
|  | *bla*_TEM_-F | TCGCCGCATACACTATTCTCAGAATGA | 50 | 445 | (42,43) |
|  | *bla*_TEM_-R | ACGCTCACCGGCTCCAGATTTAT |  |  |  |
|  | *bla*_SHV_-F | ATGCGTTATATTCGCCTGTG | 56 | 747 | (42) |
|  | *bla*_SHV_-R | TGCTTTGTTATTCGGGCCAA |  |  |  |
|  | *bla*_GES_-F | AGTCGGCTAGACCGGAAAG | 60 | 399 | (44) |
|  | *bla*_GES_-R | TTTGTCCGTGCTCAGGAT |  |  |  |
|  | *bla*_PER_-F | GCTCCGATAATGAAAGCGT | 60 | 520 | (44) |
|  | *bla*_PER_-R | TTCGGCTTGACTCGGCTGA |  |  |  |
|  | *bla*_VEB_-F | CATTTCCCGATGCAAAGCGT | 60 | 648 | (44) |
|  | *bla*_VEB_-R | CGAAGTTTCTTTGGACTCTG |  |  |  |
|  | *bla*_CTX-M-1_-F | AAAAATCACTGCGCCAGTTC | 52 | 415 | (45) |
|  | *bla*_CTX-M-1_-R | AGCTTATTCATCGCCACGTT |  |  |  |
|  | *bla*_CTX-M-9_-F | CAAAGAGAGTGCAACGGATG | 52 | 205 | (45) |
|  | *bla*_CTX-M-9_-R | ATTGGAAAGCGTTCATCACC |  |  |  |
|  | *bla*_KPC_-F | TCGCCGTCTAGTTCTGCTGTCTTG | 66 | 353 | (46) |
|  | *bla*_KPC_-R | ACAGCTCCGCCACCGTCAT |  |  |  |
| Tetracyclines | *tetA-*F | GCTACATCCTGCTTGCCTTC | 55 | 210 | (47) |
|  | *tetA-*R | CATAGATCGCCGTGAAGAGG |  |  |  |
|  | *tetB-*F | TTGGTTAGGGGCAAGTTTTG | 55 | 659 | (47) |
|  | *tetB-*R | GTAATGGGCCAATAACACCG |  |  |  |
| Sulfonamides | *sul1-F* | CTTCGATGAGAGCCGGCGGC | 65 | 436 | (48) |
|  | *sul1-R* | GCAAGGCGGAAACCCGCGCC |  |  |  |
|  | *sul2-F* | TCAACATAACCTCGGACAGT | 55 | 707 | (48) |
|  | *sul2-R* | GATGAAGTCAGCTCCACCT |  |  |  |
| Aminoglycosides | *aph(6)-Id-*F | GACTCCTGCAATCGTCAAGG | 55 | 560 | (49) |
|  | *aph(6)-Id-*R | GCAATGCGTCTAGGATCGAG |  |  |  |
|  | *aph(3")-Ib-*F | GTGGCTTGCCCCGAGGTCATCA | 55 | 612 | (50) |
|  | *aph(3")-Ib-*R | CCAAGTCAGAGGGTCCAATC |  |  |  |
|  | *aadA5-*F | CGCTCAACGCAAGATTCTCT | 55 | 792 | (51) |
|  | *aadA5-*R | ATGGGTGAATTTTTCCCTGCAC |  |  |  |
|  | *acc(6’)-Ib-cr-*F | TTGCGATGCTCTATGAGTGGCTA | 55 | 482 | (52) |
|  | *acc(6’)-Ib-cr-*R | CTCGAATGCCTGGCGTGTTT |  |  |  |
| Chloramphenicol | *catI*-F | AGTTGCTCAATGTACCTATAACC | 55 | 547 | (53) |
|  | *catI*-R | TTGTAATTCATTAAGCATTCTGCC |  |  |  |
|  | *cmIA*-F | CCGCCACGGTGTTGTTGTTATC | 55 | 698 | (53) |
|  | *cmIA*-R | CACCTTGCCTGCCCATCATTAG |  |  |  |
| Quinolones | *qnrA*-F | ATTTCTCACGCCAGGATTTG | 53 | 516 | (54) |
|  | *qnrA*-R | GATCGGCAAAGGTTAGGTCA |  |  |  |
|  | *qnrS*-F | ACGACATTCGTCAACTGCAA | 53 | 417 | (54) |
|  | *qnrS*-R | TAAATTGGCACCCTGTAGGC |  |  |  |
|  | *fla*-R | GMYTGGTTGCGRATGGT |  |  |  |
|  | *hlyA*-R | GGCGGCGCCGGACGAGACGGG |  |  |  |
| Erythromycin and clindamycin | *ermA-*F | AAGCGGTAAACCCCTCTGA | 55 | 190 | (55) |
|  | *ermA-*R | TTCGCAAATCCCTTCTCAAC |  |  |  |
|  | *ermB-*F | CTATCTGATTGTTGAAGAAGGATT | 52 | 142 | (56) |
|  | *ermB-*R | GTTTACTCTTGGTTTAGGATGAAA |  |  |  |
|  | *ermC-*F | AATCGTCAATTCCTGCATGT | 55 | 297 | (57) |
|  | *ermC-*R | TAATCGTGGAATACGGGTTTG |  |  |  |
|  | *mrsA-*F | TCCAATCATTGCACAAAATC | 54 | 163 | (58) |
|  | *mrsA-*R | AATTCCCTCTATTTGGTGGT |  |  |  |
| Rifampicin | *rpoB-*F | AGAGAGTAAGGTCCGATTGAAC | 55 | 370 | (59) |
|  | *rpoB-*R | GGTTGTTTCCCGTATTATGC |  |  |  |
| Methicillin resistance gene | *mecA-*F | GGGATCATAGCGTCATTATTC | 61 | 527 | (60) |
|  | *mecA-*R | AACGATTGTGACACGATAGCC |  |  |  |

| Location | Pilot site | Sample type | Model | N samples | N samples pre | N samples  post | Outcome | Estimate IRR | CI low | CI high | *p*-value |
| --- | --- | --- | --- | --- | --- | --- | --- | --- | --- | --- | --- |
| Valencia, Spain | Office | Human | Poisson | 46 | 24 | 22 | Isolate count | 1.6103 | 0.9254 | 2.8022 | 0.0918 |
|  |  |  |  |  |  |  | Genus richness | 1.3939 | 0.7522 | 2.5829 | 0.2912 |
|  |  |  |  |  |  |  | Taxon richness | 1.6076 | 0.8978 | 2.8787 | 0.1101 |
|  |  | Air | Poisson | 16 | 8 | 8 | Isolate count | 0.1428 | 0.0559 | 0.3646 | 0.00004 |
|  |  |  |  |  |  |  | Genus richness | 0.1250 | 0.0287 | 0.5436 | 0.0055 |
|  |  |  |  |  |  |  | Taxon richness | 0.1612 | 0.0627 | 0.4147 | 0.0001 |
| Dublin, Ireland | School | Surface | Poisson | 48 | 18 | 30 | Isolate count | 1.4000 | 0.1840 | 10.6468 | 0.7451 |
|  |  |  |  |  |  |  | Genus richness | 1.0000 | 0.1280 | 7.8120 | 1.0000 |
|  |  |  |  |  |  |  | Taxon richness | 1.0000 | 0.128 | 7.8120 | 1.0000 |
|  |  | Air | Poisson | 26 | 15 | 11 | Isolate count | 1.3804 | 0.8671 | 2.1975 | 0.1741 |
|  |  |  |  |  |  |  | Genus richness | 1.1396 | 0.6374 | 2.0376 | 0.6591 |
|  |  |  |  |  |  |  | Taxon richness | 1.3207 | 0.7974 | 2.1874 | 0.2797 |

**Supplementary Table 4.** Sample-level recovery and richness models.

IRR, incidence rate ratio; CI, confidence interval.

**Supplementary Table 5.** Jaccard PERMANOVA and PERMDISP statistics.

|  |  |  |  |  |  | PERMANOVA | | | | | PERMDISP | | |
| --- | --- | --- | --- | --- | --- | --- | --- | --- | --- | --- | --- | --- | --- |
| Location | **Pilot site** | **Sample type** | **N samples** | **N samples included in Jaccard analysis** | **Zero-isolate samples excluded** | **pseudo-F** | **R^2^** | ***p*** | **BH-adjusted *p*** | **F** | | ***p*** | **BH-adjusted *p*** |
| Valencia, Spain | Office | Human | 46 | 30 | 16 | 2.3032 | 0.0760 | 0.0311 | 0.0526 | 2.6966 | | 0.1118 | 0.2236 |
|  |  | Air | 16 | 8 | 8 | 1.7812 | 0.2289 | 0.0738 | 0.0738 | 102.98 | | 0.0001 | 0.0004 |
| Dublin, Ireland | School | Surface | 48 | 43 | 5 | 3.9150 | 0.0871 | 0.0011 | 0.0044 | 1.6074 | | 0.2105 | 0.2735 |
|  |  | Air | 26 | 21 | 5 | 1.7756 | 0.0854 | 0.0395 | 0.0526 | 1.2711 | | 0.2735 | 0.2735 |

**Supplementary Table 6.** Phenotypic resistance effect estimates.

|  |  |  | Samples pre | | Samples post | | Prevalence | |  |  |  |  |
| --- | --- | --- | --- | --- | --- | --- | --- | --- | --- | --- | --- | --- |
| Location | **Pilot site** | **Sample type** | **N total** | **N positive** | **N total** | **N positive** | **Pre** | **Post** | **RR post/pre** | **RR CI low** | **RR CI high** | **Fisher *p*** |
| Valencia, Spain | Office | Human | 17 | 2 | 31 | 6 | 0.1176 | 0.1935 | 1.6451 | 0.3718 | 7.2778 | 0.6938 |
|  |  | Air | 37 | 9 | 5 | 0 | 0.2432 | 0.0000 | 0.3333 | 0.0221 | 5.0117 | 0.5670 |
| Dublin, Ireland | School | Surface | 51 | 22 | 43 | 9 | 0.4313 | 0.2093 | 0.4852 | 0.2505 | 0.9396 | 0.0283 |
|  |  | Air | 29 | 7 | 45 | 8 | 0.2413 | 0.1777 | 0.7365 | 0.2992 | 1.8126 | 0.5615 |

RR, risk ratio; CI, confidence interval.

**Supplementary Table 7.** Antimicrobial susceptibility profiles of culturable bacterial isolates from office human swab samples before and after portable air purifier deployment. Resistant (R; red); Intermediate (I; orange); Susceptible (S; green); antibiotic not tested (grey).

| **Phase** | **Species** | **AMP** | **AMC** | **TZP** | **FOX** | **CTX** | **CAZ** | **FEP** | **IMI** | **MEM** | **ATM** | **AN** | **GM** | **CIP** | **CHL** | **TET** | **SXT** | **RIF** | **CC** | **ARGs** |
| --- | --- | --- | --- | --- | --- | --- | --- | --- | --- | --- | --- | --- | --- | --- | --- | --- | --- | --- | --- | --- |
| **Pre** | *A. ursingii* |  |  | S |  | I | I | S | S | S |  | S | S | S |  | S | S |  |  | *-* |
|  | *S. marcescens* | R | R | S |  | S | S | S | S | S |  | S | S | S | S | R | S |  |  | *-* |
|  | *Pseudomonas* spp. |  |  | S |  |  | S | S | S | S | I | S |  | S |  |  |  |  |  | *-* |
| **Post-intervention** | *A. ursingii* |  |  | S |  | S | S | S | S | I |  | S | S | S |  | S | S |  |  | *-* |
|  | *S. marcescens* | R | R | S |  | S | S | S | S | S |  | S | S | S | S | R | S |  |  | *-* |
|  | *P. luteola* |  |  | S |  |  | S | S | S | S | I | S |  | I |  |  |  |  |  | *-* |
|  | *P. luteola* |  |  | S |  |  | S | S | S | S | I | S |  | S |  |  |  |  |  | *-* |
|  | *P. oryzihabitans* |  |  | S |  |  | S | S | S | S | R | S |  | S |  |  |  |  |  | *-* |
|  | *S. epidermidis* |  |  |  | R |  |  |  |  |  |  |  | S | S | S | R | S | S | S | *-* |
|  | *S. epidermidis* |  |  |  | S |  |  |  |  |  |  |  | R | S | S | S | S | S | S | *-* |
|  | *S. epidermidis* |  |  |  | R |  |  |  |  |  |  |  | S | S | S | R | S | R | R | *ermA* |

Amikacin (AN), amoxicillin/clavulanic acid (AMC), ampicillin (AMP), aztreonam (ATM), cefepime (FEP), cefoxitin (FOX), cefotaxime (CTX), ceftazidime (CAZ), chloramphenicol (CHL), ciprofloxacin (CIP), clindamycin (CC), gentamicin (GM), imipenem (IMI), meropenem (MEM), piperacillin–tazobactam (TZP), rifampicin (RIF), tetracycline (TET), trimethoprim/sulfamethoxazole (SXT).

**Supplementary Table 8.** Antimicrobial susceptibility profiles of culturable bacterial isolates from office air samples before and after portable air purifier deployment. Resistant (R; red); Intermediate (I; orange); Susceptible (S; green); antibiotic not tested (grey).

| **Phase** | **Species** | **TZP** | **FOX** | **CAZ** | **FEP** | **IMI** | **MEM** | **ATM** | **AN** | **GM** | **CIP** | **CHL** | **TET** | **SXT** | **RIF** | **CC** | **E** | **ARGs** |
| --- | --- | --- | --- | --- | --- | --- | --- | --- | --- | --- | --- | --- | --- | --- | --- | --- | --- | --- |
| **Pre-intervention** | *M. luteus* |  |  |  |  |  |  |  |  |  |  |  |  |  |  | I | R | - |
|  | *M. luteus* |  |  |  |  |  |  |  |  |  |  |  |  |  |  | S | R | - |
|  | *M. luteus* |  |  |  |  |  |  |  |  |  |  |  |  |  |  | S | R | - |
|  | *P. luteola* | S |  | S | S | S | S | I | S |  | S |  |  |  |  |  |  | - |
|  | *Pseudomonas* spp. | S |  | S | S | S | S | I | S |  | S |  |  |  |  |  |  | - |
|  | *S. hominis* |  | S |  |  |  |  |  |  | S | S | S | R | S | S | S |  | - |
|  | *S. hominis* |  | R |  |  |  |  |  |  | S | S | S | S | R | S | R |  | *ermC, mecA* |
|  | *S. hominis* |  | R |  |  |  |  |  |  | S | S | S | S | S | S | S |  | *mecA* |
|  | *S*. *succinus* |  | R |  |  |  |  |  |  | S | S | S | S | S | S | I |  | *mecA* |
|  | *S. warneri* |  | S |  |  |  |  |  |  | S | S | S | R | S | S | S |  | - |
|  | *S. xylosus* |  | S |  |  |  |  |  |  | S | S | S | S | S | S | I |  | - |

Amikacin (AN), aztreonam (ATM), cefepime (FEP), cefoxitin (FOX), ceftazidime (CAZ), chloramphenicol (CHL), ciprofloxacin (CIP), clindamycin (CC), erythromycin (E), gentamicin (GM), imipenem (IMI), meropenem (MEM), piperacillin–tazobactam (TZP), rifampicin (RIF), tetracycline (TET), trimethoprim/sulfamethoxazole (SXT).

**Supplementary Table 9.** Antimicrobial susceptibility profiles of culturable bacterial isolates from school surface samples before and after portable air purifier deployment. Resistant (R; red); Intermediate (I; orange); Susceptible (S; green); antibiotic not tested (grey).

| **Phase** | **Species** | **AMP** | **AMC** | **TZP** | **FOX** | **CTX** | **CAZ** | **FEP** | **IMI** | **MEM** | **ATM** | **AN** | **GM** | **CIP** | **CHL** | **TET** | **SXT** | **RIF** | **CC** | **E** | **ARGs** |
| --- | --- | --- | --- | --- | --- | --- | --- | --- | --- | --- | --- | --- | --- | --- | --- | --- | --- | --- | --- | --- | --- |
| **Pre-intervention** | *A. viridans* | S |  |  |  |  |  |  |  | R |  |  |  | R |  |  |  | R |  |  | - |
|  | *B. cereus* |  |  |  |  |  |  |  | R | S |  |  |  | I |  |  |  |  | S | S | - |
|  | *Bacillus* spp. |  |  |  |  |  |  |  | S | S |  |  |  | I |  |  |  |  | S | S | - |
|  | *Bacillus* spp. |  |  |  |  |  |  |  | R | R |  |  |  | I |  |  |  |  | S | S | - |
|  | *Bacillus* spp. |  |  |  |  |  |  |  | S | S |  |  |  | I |  |  |  |  | R | R | - |
|  | *Bacillus* spp. |  |  |  |  |  |  |  | S | S |  |  |  | I |  |  |  |  | S | S | - |
|  | *Bacillus* spp*.* |  |  |  |  |  |  |  | S | S |  |  |  | I |  |  |  |  | S | S | - |
|  | *P. agglomerans* | S | S | R | S | S | S | I | S | R |  |  | I | R | I | S | R |  |  |  | - |
|  | *P. agglomerans* | S | S | S | S | S | S | S | S | S |  |  | S | I | S | S | R |  |  |  | - |
|  | *P. agglomerans* | S | S | S | S | S | S | S | I | R |  |  | S | R | I | R | R |  |  |  | - |
|  | *P. agglomerans* | S | S | S | S | S | S | S | R | R |  |  | S | I | S | S | R |  |  |  | - |
|  | *P. agglomerans* | S | S | S | S | S | S | S | I | R |  |  | S | S | S | S | R |  |  |  | - |
|  | *P. septica* | S | I | R | R | R | S | I | S | R |  |  | S | S | S | S | R |  |  |  | - |
|  | *P. septica* | S | I | R | R | R | S | I | S | R |  |  | I | R | I | S | R |  |  |  | - |
|  | *P. septica* | S | S | R | R | R | R | I | S | I |  |  | S | S | S | S | R |  |  |  | - |
|  | *Pantoea* spp. | S | S | I | S | R | S | I | S | S |  |  | S | I | S | S | R |  |  |  | - |
|  | *Pantoea* spp. | S | S | S | S | S | S | S | S | S |  |  | S | I | S | S | R |  |  |  | - |
|  | *P. fulva* |  |  | S |  |  | S | S | S | S | I | S |  | S |  |  |  |  |  |  | - |
|  | *P. koreensis* |  |  | S |  |  | S | S | S | S | R | S |  | S |  |  |  |  |  |  | - |
|  | *P. oryzihabitans* |  |  | S |  |  | S | S | S | S | I | S |  | S |  |  |  |  |  |  | - |
|  | *Pseudomonas* spp. |  |  | S |  |  | S | S | S | S | I | S |  | S |  |  |  |  |  |  | - |
|  | *Pseudomonas* spp. |  |  | S |  |  | S | S | S | S | R | S |  | S |  |  |  |  |  |  | - |
|  | *S. haemolyticus* |  |  |  | R |  |  |  |  |  |  |  | S | S | S | S | R | S | S |  | *mecA* |
|  | *S. saprophyticus* |  |  |  | S |  |  |  |  |  |  |  | S | S | S | S | R | S | S |  | - |
|  | *S. saprophyticus* |  |  |  | S |  |  |  |  |  |  |  | S | S | S | S | R | S | S |  | - |
|  | *S. simulans* |  |  |  | S |  |  |  |  |  |  |  | S | S | S | S | R | S | S |  | - |
| **Post-intervention** | *A. viridans* | S |  |  |  |  |  |  |  | S |  |  |  | S |  |  |  | R |  |  | - |
|  | *B. licheniformis* |  |  |  |  |  |  |  | S | S |  |  |  | I |  |  |  |  | R | R | - |
|  | *P. agglomerans* | S | S | S | S | R | S | S | S | S |  |  | S | S | S | S | S |  |  |  | - |
|  | *P. chlororaphis* |  |  | I |  |  | S | S | S | S | S | S |  | S |  |  |  |  |  |  | - |
|  | *P. fulva* |  |  | S |  |  | S | S | S | S | I | S |  | S |  |  |  |  |  |  | - |
|  | *P. putida* |  |  | S |  |  | S | S | S | S | I | S |  | S |  |  |  |  |  |  | - |
|  | *P. rhodesiae* |  |  | S |  |  | S | S | S | S | R | S |  | S |  |  |  |  |  |  | - |
|  | *S. liquefaciens* | R | R | I |  | S | S | S | S | S |  | S | S | S | S | S | S |  |  |  | - |
|  | *S. parasanguinis* |  |  |  | S |  |  |  |  |  |  |  | S | S | S | R | S | S | S |  | - |

Amikacin (AN), amoxicillin/clavulanic acid (AMC), ampicillin (AMP), aztreonam (ATM), cefepime (FEP), cefoxitin (FOX), cefotaxime (CTX), ceftazidime (CAZ), chloramphenicol (CHL), ciprofloxacin (CIP), clindamycin (CC), erythromycin (E), gentamicin (GM), imipenem (IMI), meropenem (MEM), piperacillin–tazobactam (TZP), rifampicin (RIF), tetracycline (TET), trimethoprim/sulfamethoxazole (SXT).

**Supplementary Table 10.** Antimicrobial susceptibility profiles of culturable bacterial isolates from school air samples before and after portable air purifier deployment. Resistant (R; red); Intermediate (I; orange); Susceptible (S; green); antibiotic not tested (grey).

| **Phase** | **Species** | **AMP** | **AMC** | **TZP** | **FOX** | **CTX** | **CAZ** | **FEP** | **IMI** | **MEM** | **ATM** | **AN** | **GM** | **CIP** | **CHL** | **TET** | **SXT** | **RIF** | **CC** | **E** | **ARGs** |
| --- | --- | --- | --- | --- | --- | --- | --- | --- | --- | --- | --- | --- | --- | --- | --- | --- | --- | --- | --- | --- | --- |
| **Pre-intervention** | *A. johnsonii* |  |  | S |  | I | S | S | S | S |  | S | S | S |  | S | S |  |  |  | - |
|  | *A. viridans* | S |  |  |  |  |  |  |  | S |  |  |  | R |  |  |  | S |  |  | - |
|  | *A. viridans* | S |  |  |  |  |  |  |  | R |  |  |  | R |  |  |  | S |  |  | - |
|  | *A. viridans* | S |  |  |  |  |  |  |  | S |  |  |  | S |  |  |  | R |  |  | - |
|  | *A. viridans* | S |  |  |  |  |  |  |  | R |  |  |  | R |  |  |  | R |  |  | - |
|  | *A. viridans* | S |  |  |  |  |  |  |  | R |  |  |  | R |  |  |  | R |  |  | - |
|  | *B. weihenstephanensis* |  |  |  |  |  |  |  | S | S |  |  |  | I |  |  |  |  | S | S | - |
|  | *B. weihenstephanensis* |  |  |  |  |  |  |  | S | S |  |  |  | I |  |  |  |  | S | S | - |
|  | *M. luteus* |  |  |  |  |  |  |  |  |  |  |  |  |  |  |  |  |  | S | R | - |
|  | *P. agglomerans* | S | I | S | S | I | I | S | S | S |  |  | S | S | S | S | S |  |  |  | - |
|  | *P. eucrina* | S | S | S | S | S | I | S | I | S |  |  | S | S | S | S | R |  |  |  | - |
|  | *P. septica* | S | I | S | S | R | I | R | S | S |  |  | S | S | S | S | S |  |  |  | - |
|  | *P. septica* | S | I | S | S | R | I | I | I | R |  |  | S | I | I | S | I |  |  |  | - |
|  | *P. septica* | S | I | S | S | R | S | R | I | S |  |  | S | S | S | S | S |  |  |  | - |
|  | *P. septica* | S | S | S | S | I | S | S | S | I |  |  | S | S | S | S | S |  |  |  | - |
|  | *P. septica* | S | S | S | S | I | S | S | S | S |  |  | S | S | S | S | S |  |  |  | - |
|  | *P. koreensis* |  |  | S |  |  | S | S | S | S | R | S |  | S |  |  |  |  |  |  | - |
|  | *P. luteola* |  |  | S |  |  | S | S | S | S | I | S |  | S |  |  |  |  |  |  | - |
|  | *S. cohnii* |  |  |  | R |  |  |  |  |  |  |  | S | S | S | S | S | S | S |  | - |
|  | *S. epidermidis* |  |  |  | R |  |  |  |  |  |  |  | S | S | S | S | S | S | S |  | *mecA* |
|  | *S. epidermidis* |  |  |  | R |  |  |  |  |  |  |  | S | S | S | S | S | S | S |  | *mecA* |
|  | *S. hominis* |  |  |  | R |  |  |  |  |  |  |  | S | S | S | S | S | S | S |  | - |
|  | *S. hominis* |  |  |  | R |  |  |  |  |  |  |  | S | S | S | R | R | S | S |  | *mecA* |
|  | *S. saprophyticus* |  |  |  | R |  |  |  |  |  |  |  | S | S | S | S | S | S | S |  | - |
|  | *S. saprophyticus* |  |  |  | R |  |  |  |  |  |  |  | S | S | S | S | S | S | S |  | - |
|  | *S. simulans* |  |  |  | S |  |  |  |  |  |  |  | S | S | S | R | S | S | S |  | - |
|  | *S. succinus* |  |  |  | R |  |  |  |  |  |  |  | S | S | S | S | S | S | S |  | - |
| **Post-intervention** | *P. agglomerans* | I | I | I | S | S | S | S | S | S |  |  | S | S | S | S | S |  |  |  | - |
|  | *P. anthophila* | R | S | I | S | S | S | S | S | S |  |  | S | S | S | S | S |  |  |  | - |
|  | *P. septica* | R | S | R | S | S | S | S | S | S |  |  | S | S | S | S | S |  |  |  | - |
|  | *P. septica* | R | S | S | R | S | S | S | S | S |  |  | S | S | S | S | S |  |  |  | - |
|  | *P. septica* | R | S | I | S | S | S | S | S | S |  |  | S | S | S | S | S |  |  |  | - |
|  | *Pantoea* spp*.* | R | R | S | S | S | S | S | S | S |  |  | S | S | S | S | S |  |  |  | - |
|  | *Pseudomonas* spp*.* |  |  | I |  |  | S | S | S | S | I | S |  | S |  |  |  |  |  |  | - |
|  | *S. epidermidis* |  |  |  | R |  |  |  |  |  |  |  | S | S | S | S | S | S | S |  | - |
|  | *S. haemolyticus* |  |  |  | R |  |  |  |  |  |  |  | S | S | S | S | S | S | S |  | - |

Amikacin (AN), amoxicillin/clavulanic acid (AMC), ampicillin (AMP), aztreonam (ATM), cefepime (FEP), cefoxitin (FOX), cefotaxime (CTX), ceftazidime (CAZ), chloramphenicol (CHL), ciprofloxacin (CIP), clindamycin (CC), erythromycin (E), gentamicin (GM), imipenem (IMI), meropenem (MEM), piperacillin–tazobactam (TZP), rifampicin (RIF), tetracycline (TET), trimethoprim/sulfamethoxazole (SXT).

# 2 References

41. Owusu FA, Obeng-Nkrumah N, Gyinae E, Kodom S, Tagoe R, Tabi BKA, et al. Occurrence of Carbapenemases, Extended-Spectrum Beta-Lactamases and AmpCs among Beta-Lactamase-Producing Gram-Negative Bacteria from Clinical Sources in Accra, Ghana. Antibiotics. 2023; 12(6):1016. doi:10.3390/antibiotics12061016

42. Monstein HJ, Östholm-Balkhed Å, Nilsson M V., Nilsson M, Dornbusch K, Nilsson LE. Multiplex PCR amplification assay for the detection of blaSHV, blaTEM and blaCTX-M genes in Enterobacteriaceae. APMIS. 2007; 115(12):1400–8. doi:10.1111/J.1600-0463.2007.00722.x

43. Govender R, Amoah ID, Adegoke AA, Singh G, Kumari S, Swalaha FM, et al. Identification, antibiotic resistance, and virulence profiling of Aeromonas and Pseudomonas species from wastewater and surface water. Environ Monit Assess. 2021; 193(5):294. doi:10.1007/s10661-021-09046-6

44. Dallenne C, da Costa A, Decré D, Favier C, Arlet G. Development of a set of multiplex PCR assays for the detection of genes encoding important β-lactamases in Enterobacteriaceae. Journal of Antimicrobial Chemotherapy. 2010; 65(3):490–5. doi:10.1093/jac/dkp498

45. Woodford N, Fagan EJ, Ellington MJ. Multiplex PCR for rapid detection of genes encoding CTX-M extended-spectrum beta-lactamases. Journal of Antimicrobial Chemotherapy. 2006; 57(1):154–5. doi:10.1093/jac/dki412

46. Bispo dos Santos S, Fernandez Alarcon M, Stella Ballaben A, Harakava R, Galetti R, Cardoso Guimarães M, et al. First report of Aeromonas veronii as an emerging bacterial pathogen of farmed nile tilapia (Oreochromis niloticus) in Brazil. Pathogens. 2023; 12(8):1020. doi:10.3390/pathogens12081020

47. Ramírez-Bayard IE, Mejía F, Medina-Sánchez JR, Cornejo-Reyes H, Castillo M, Querol-Audi J, et al. Prevalence of plasmid-associated tetracycline resistance genes in multidrug-resistant Escherichia coli strains isolated from environmental, animal and human samples in panama. Antibiotics. 2023; 12:280. doi:10.3390/ antibiotics12020280

48. Guerra B, Junker E, Miko A, Helmuth R, Mendoza MC. Characterization and Localization of Drug Resistance Determinants in Multidrug-Resistant, Integron-Carrying Salmonella enterica Serotype Typhimurium Strains. Microbial Drug Resistance. 2004; 10(2):83–91. doi:10.1089/1076629041310136

49. Overbeek LS, Wellington EMH, Egan S, Smalla K, Heuer H, Collard JM, et al. Prevalence of streptomycin-resistance genes in bacterial populations in European habitats. FEMS Microbiol Ecol. 2006; 42(2):277–88. doi:10.1111/j.1574-6941.2002.tb01018.x

50. Miró E, Grünbaum F, Gómez L, Rivera A, Mirelis B, Coll P, et al. Characterization of aminoglycoside-modifying enzymes in Enterobacteriaceae clinical strains and characterization of the plasmids implicated in their diffusion. Microbial Drug Resistance. 2013; 19(2):94–9. doi:10.1089/mdr.2012.0125

51. Liang H, Li X, Yan H. Identification of a novel IncHI1B plasmid in MDR Klebsiella pneumoniae 200 from swine in China. Antibiotics. 2022; 11(9):1225. doi:10.3390/antibiotics11091225

52. Tahbaz S V., Azimi L, Lari AR. Characterization of aminoglycoside resistance mechanisms in acinetobacter baumannii isolates from burn wound colonization. Ann Burns Fire Disasters. 2019; 32(2):115–21. PubMed PMID: 31528151

53. Torkan S, Bahadoranian MA, Khamesipour F, Anyanwu MU. Detection of virulence and antimicrobial resistance genes in Escherichia coli isolates from diarrhoiec dogs in Iran. Arch Med Vet. 2016; 48(2):181–90. doi:10.4067/S0301-732X2016000200008

54. Wang A, Yang Y, Lu Q, Wang Y, Chen Y, Deng L, et al. Presence of qnr gene in Escherichia coli and Klebsiella pneumoniae resistant to ciprofloxacin isolated from pediatric patients in China. BMC Infect Dis. 2008; 8:68. doi:10.1186/1471-2334-8-68

55. Timsina R, Shrestha U, Singh A, Timalsina B. Inducible clindamycin resistance and erm genes in Staphylococcus aureus in school children in Kathmandu, Nepal. Future Sci OA. 2021; 7(1):FSO361. doi:10.2144/fsoa-2020-0092

56. Shaaban F, Salem Sokhn E, Khalil C, Saleh FA. Antimicrobial activity of adipose-derived mesenchymal stromal cell secretome against methicillin-resistant Staphylococcus aureus. Stem Cell Res Ther. 2025; 16(1):21. doi:10.1186/s13287-025-04138-3

57. Boyanov VS, Alexandrova AS, Hristova PM, Hitkova HY, Gergova RT. Antibiotic Resistance and Serotypes Distribution in Streptococcus agalactiae Bulgarian Clinical Isolates During the Years of 2021–2024. Pol J Microbiol. 2024; 73(4):505–14. doi:10.33073/pjm-2024-042

58. Tareen AR, Zahra R. Community acquired methicillin resistant Staphylococci (CA-MRS) in fecal matter of wild birds – A ‘one health’ point of concern. J Infect Public Health. 2023; 16(6):877–83. doi:10.1016/j.jiph.2023.04.002

59. Shbibe L, Battah B, Kassab YW, Qureshi MJ, Al-Temimi AA, Soukkarieh C, et al. PCR identification of Enterococcus Species as a Primary Pathogen in Neonatal Bloodstream Infections at Damascus University Paediatric Hospital. Res J Pharm Technol. 2024; 17(8):3997–4001. doi:10.52711/0974-360X.2024.00620

60. Ruzauskas M, Siugzdiniene R, Klimiene I, Virgailis M, Mockeliunas R, Vaskeviciute L, et al. Prevalence of methicillin-resistant Staphylococcus haemolyticus in companion animals: a cross-sectional study. Ann Clin Microbiol Antimicrob. 2014; 13(1):56. doi:10.1186/s12941-014-0056-y
